# C-peptide Trajectory Following Pancreas Transplantation

**DOI:** 10.7759/cureus.80103

**Published:** 2025-03-05

**Authors:** Jordan A Williamson, Kayla J Dobies, Abraham M Velazquez, Oliver G Ralph, Oyedolamu Olaitan

**Affiliations:** 1 Surgery, Rush University Medical Center, Chicago, USA

**Keywords:** c-peptide, glomerular filtration rate, pancreas transplantation, patient outcome assessment, type 2 diabetes mellitus

## Abstract

Background: Pancreas transplantation is the most reliable management of insulin-dependent diabetes mellitus, offering sustained glycemic control with a reduction in diabetes-related complications. Despite recent advancements, recipient selection criteria are not standardized. Historically, pre-transplant C-peptide level was an important indicator of post-transplant success, yet conflicting data exist regarding their correlation. This study explores post-transplant C-peptide trends in recipients with varying pre-transplant C-peptide levels, aiming to elucidate its impact on patient and graft survival.

Methods: A retrospective review of 78 pancreas transplant recipients (simultaneous pancreas and kidney, pancreas after kidney, and pancreas transplant alone) from September 2012 to August 2022 was conducted. Patients were categorized based on pre-transplant C-peptide levels (>4.0 ng/mL elevated vs. ≤4.0 ng/mL low/normal). C-peptide levels, HbA1c, and estimated glomerular filtration rate (eGFR) were monitored at specified intervals post-transplant.

Results: The two cohorts exhibited disparate post-transplant C-peptide trends; elevated (pre-transplant: mean = 8.43 ng/mL, range = 4-28.26 ng/mL; post-transplant: mean = 3.57 ng/mL, range = 0.84-8.53 ng/mL) and low/normal (pre-transplant: mean = 1.07 ng/mL, range = 0-3.92 ng/mL; post-transplant: mean = 2.81 ng/mL, range = 0.9-6.73 ng/mL). Despite achieving normoglycemic control (HbA1c 5.26% and 5.19%, respectively), the decline in C-peptide levels in the elevated pre-transplant group contradicted the anticipated outcomes.

Conclusion: This study highlights the intricate dynamics of post-transplant C-peptide, revealing unexpected patterns in recipients with elevated pre-transplant C-peptide levels. The study's findings question the predictive value of pre-transplant C-peptide levels and underscore the importance of further research to unravel its metabolism post-transplant.

## Introduction

Pancreas transplantation is the most reliable treatment for insulin-dependent diabetes mellitus, providing long-term control of diabetes without the need for exogenous insulin and preventing secondary complications of the disease [1-3]. Despite numerous advancements in the field in recent years, recipient selection factors are not standardized. Historically, pre-transplant C-peptide level was one of the most important predictors for post-transplant success in pancreas transplant recipients. Conflicting data has been presented, illustrating low/normal (≤4.0 ng/mL) versus elevated (>4.0 ng/mL) pre-transplant C-peptide levels correlating or not correlating with post-transplant success [4-6]. Recent studies have shown an association between elevated pre-transplant C-peptide and complication rates such as graft failure, the development of post-transplant diabetes mellitus, and increased mortality rates [7-10]. However, no study has focused on post-transplant C-peptide trends in those with low/normal versus elevated pre-transplant C-peptide, its significance on patient and graft survival, and how it factors into our understanding of insulin metabolism post-transplant.

We present a single-center retrospective review of 78 patients who received a pancreas transplant - simultaneous pancreas and kidney (SPK), pancreas after kidney (PAK), or pancreas transplant alone (PTA) over a 10-year period. We hypothesized that those with low/normal and elevated C-peptide levels pre-transplant would have similar success rates post-transplant and that post-transplant C-peptide levels would increase for all groups, considering the increased insulin production from the nascent pancreas.

We aimed to understand post-transplant C-peptide trends in comparison to pre-transplant levels. We compared post-transplant kidney function between the two groups as a possible confounder, as insulin and C-peptide are largely cleared from systemic circulation by the kidney.

## Materials and methods

This was a single-center retrospective study comparing all patients who underwent pancreas, with or without a kidney transplant, from September 2012 to August 2022 with low/normal (≤4.0 ng/mL) and elevated (>4.0 ng/mL) pre-transplant C-peptide at a single institution. A total of 78 patients were included in the analysis. The study was approved by the institutional review board.

Patients were eligible for SPK or PAK transplantation if they met the following criteria: the patient was insulin dependent with C-peptide ≤2 ng/mL or there was evidence of autoimmune diabetes [11]. If C-peptide was >2 ng/mL, or there was no evidence of autoimmune diabetes, then they must have a body mass index (BMI) ≤30 kg/m^2^ and a total daily insulin requirement of <1.0 unit/kg/day [11]. For PTA, they had to have type 1 diabetes with undetectable C-peptide or evidence of autoimmune diabetes.

Electronic medical records (EMRs) were used to conduct chart reviews with a minimum follow-up of one year. Fasting C-peptide levels were collected on the day of transplant (time 0) and at 1, 3, 6, and 12 months post-transplant. Our institution’s lab defined an elevated C-peptide as >4.0 ng/mL. HbA1c levels were also collected on the day of transplant (time 0) and at 3, 6, 9, and 12 months post-transplant. Glycemic control was defined as an HbA1c ≤5.7%. Estimated glomerular filtration rate (eGFR) was collected for all patients on the day of transplant (time 0) and at 3, 6, 9, and 12 months post-transplant. eGFR intervals were based on the Kidney Disease Outcomes Quality Initiative (KDQI) Chronic Kidney Disease (CKD) Staging Parameters set forth by the National Kidney Foundation [12]. Due to lab discrepancies in reporting, the highest possible collective interval for this data set was ≥60 mL/min.

Statistical methods

Categorical data were presented as percentage frequencies, with continuous data presented as mean ± SD. Categorical data were analyzed using the chi-square test or Fisher's exact test, as appropriate. Continuous data were analyzed using the two-sample t-test or Mann-Whitney U test, as appropriate. Sensitivity analysis was done by performing univariate tests using data from the patients who received an SPK. Multiple mixed effect models and linear regression models were used to determine the group difference while adjusting for GFR. Two-tailed tests were used. Statistical significance was defined as p < 0.05. Analyses were performed with Statistical Analysis System (SAS) (version 9.4, SAS Institute Inc., Cary, NC).

## Results

Basic demographic data were comparable between the two cohorts at the time of transplant (Table 1). Seventy-two patients had SPK transplants, and six underwent either PAK or PTA transplants within the 10-year review period. Fifty-eight patients had low/normal pre-transplant C-peptide levels, mean 1.07 ng/mL (range: 0-3.92 ng/mL); 20 patients had elevated pre-transplant levels, mean 8.43 ng/mL (range: 4.01-28.26 ng/mL). The low/normal cohort experienced an overall increase, mean 2.81 ng/mL (range: 0.9-6.73 ng/mL) at each measured time point post-transplant, whereas the elevated cohort experienced a decline, mean 3.57 ng/mL (range: 0.84-8.53 ng/mL) at all time points post-transplant up to one year (Table 2). Of the 18 SPK patients with elevated pre-transplant C-peptide, 12 (66%) of them had a decrease at one year post-transplant, while only three (5%) of the 54 SPK patients with low/normal pre-transplant C-peptide had a decrease in their C-peptide at one year. Both C-peptide groups had HbA1c levels within normal glycemic range at all time points within the one-year post-transplant follow-up period. This was also true when SPK patients were analyzed alone (Table 2). C-peptide at month 9 and HbA1c at month 1 were left blank as those data were not routinely collected at those time points.

**Table 1:** Summary of all patient demographics at the time of transplant.

	C-peptide pre-transplant ≤ 4 ng/mL (N=58), number of patients	C-peptide pre-transplant > 4 ng/mL (N=20), number of patients
Age (years) (standard deviation)	45.34 ± 10.12	51.96 ± 7
BMI (kg/m^2^) (standard deviation)	26.39 ± 3.47	26.13 ± 4.36
Weight (kg) (standard deviation)	77.81 ± 14.17	76.71 ± 14.43
Duration of diabetes (years) (standard deviation)	23.12 ± 8.91	18.45 ± 9.58
Black individuals	26	5
Hispanic/Latino individuals	10	10
White individuals	19	2
Others	3	3

**Table 2:** Summary of C-peptide and HbA1c comparing all patients and SPK patients only up to 12 months post-transplant. SPK: simultaneous pancreas and kidney

Time After Transplant (months)	All patients, C-peptide (ng/mL)			All patients, HbA1c (%)			SPK patients, C-peptide (ng/mL)			SPK patients, HbA1c (%)		
	C-peptide pre-transplant ≤ 4 ng/mL (N=58) mean (standard deviation)	C-peptide pre-transplant > 4 ng/mL (N=20) mean (standard deviation)	P-value (<0.05)	C-peptide pre-transplant ≤ 4 ng/mL (N=58) mean (standard deviation)	C-peptide pre-transplant > 4 ng/mL (N=20) mean (standard deviation)	P-value (<0.05)	C-peptide pre-transplant ≤ 4 ng/mL (N=53) mean (standard deviation)	C-peptide pre-transplant > 4 ng/mL (N=18) mean (standard deviation)	P-value (<0.05)	C-peptide pre-transplant ≤ 4 ng/mL (N=53) mean (standard deviation)	C-peptide pre-transplant > 4 ng/mL (N=18) mean (standard deviation)	P-value (<0.05)
0	1.07 ± 1.26	8.43 ± 5.91	-	7.69 ± 1.46	7.51 ± 1.39	0.7056	1.12 ± 1.27	8.79 ± 6.12	-	7.63 ± 1.44	7.32 ± 1.31	0.475
1	3.96 ± 2.85	5.56 ± 2.66	0.0069	-	-	-	3.95 ± 2.85	5.69 ± 2.80	0.0089	-	-	-
3	2.74 ± 1.31	4.36 ± 2.17	0.0006	4.91 ± 0.55	4.76 ± 0.46	0.3404	2.72 ± 1.32	4.53 ± 2.24	0.0005	4.90 ± 0.56	4.73 ± 0.48	0.2645
6	3.08 ± 1.77	3.71 ± 1.34	0.02	5.19 ± 1.05	5.28 ± 1.03	0.8928	3.07 ± 1.80	3.67 ± 1.41	0.0361	5.22 ± 1.07	5.29 ± 1.10	1
9	-	-	-	6.41 ± 2.13	5.23 ± 0.38	0.4466	-	-	-	6.41 ± 2.13	5.23 ± 0.38	0.4466
12	2.81 ± 1.29	3.57 ± 1.92	0.2038	5.16 ± 1.03	5.26 ± 0.61	0.3795	2.72 ± 1.25	3.43 ± 1.88	0.2295	5.18 ± 1.06	5.17 ± 0.57	0.6628

We ran a separate analysis involving SPK patients alone. Fifty-four of these patients fell within the low/normal cohort, mean 1.12 ng/mL (range: 0-3.92 ng/mL); 18 patients were in the elevated cohort, mean 8.79 ng/mL (range: 4.01-28.26 ng/mL). Univariate analysis showed the same trends in the SPK sensitivity analysis that were seen in all patients. The low/normal cohort had an overall increase at each relevant time point post-transplant, mean 2.72 ng/mL (range: 0.9-6.73 ng/mL), and the elevated cohort had an overall decline, mean 3.43 ng/mL (range: 0.84-8.53 ng/mL), in their C-peptide at one year (Table 2).

The eGFR for both cohorts, with respect to all patients and SPK patients alone, is summarized in Table 3. For all patients, there was an expected increase in eGFR at three months post-transplant. This was maintained until month 12, with most patients in both cohorts falling into normal to mildly decreased CKD categories (Table 3). It is important to note that eGFR for both cohorts mostly remained within stages 1 and 2 CKD in the 12-month time frame for all patients and SPK patients alone. Multivariable regression analysis was performed and found no difference in C-peptide levels while adjusting for eGFR between SPK and PKA/PTA in the mixed effect model (β = -0.3622, standard error (SE) = 0.55, p = 0.5139). In the univariate analysis run to observe SPK patients alone, there was also an improvement in eGFR throughout the study period. There was a significant difference between eGFR at month 9 and the C-peptide cohorts (p = 0.0045). This difference was not seen in the total population and is likely explained by the presence of two patients in the elevated cohort for all patients with an eGFR between 30 and 59, whereas no SPK patients in the elevated cohort fell within this range (Table 3).

**Table 3:** Distribution of all patients and SPK patients across chronic kidney disease stages based on eGFR, comparing C-peptide cohorts up to 12 months post-transplant SPK: simultaneous pancreas and kidney; eGFR: estimated glomerular filtration rate; KDQI: Kidney Disease Outcomes Quality Initiative

Time after transplant (months)	KDQI Chronic Kidney Disease Stage (%)	All patients, C-peptide pre-transplant ≤ 4 ng/mL (N=58) (mean)	All patients, C-peptide pre-transplant > 4 ng/mL (N=20) (mean)	P-value (<0.05)	SPK patients, C-peptide pre-transplant ≤ 4 ng/mL (N=54) (mean)	SPK patients, C-peptide pre-transplant > 4 ng/mL (N=18) (mean)	P-value (<0.05)
								0	<15	87.93	85	0.8714	94.44	94.44	1
15-29	5.17	5	5.56	5.56											
30-59	5.17	10	0	0											
≥60	1.72	0	0	0											
3	<15	0	0	0.6739	0	0	0.207
	15-29	1.72	0		0	0	
	30-59	29.31	20		29.63	11.11	
	≥60	68.97	80		70.37	88.89	
6	<15	3.45	0	0.6751	3.7	0	0.506
	15-29	5.17	0		5.56	0	
	30-59	27.59	20		27.78	16.67	
	≥60	63.79	80		62.96	83.33	
9	<15	0	0	0.0507	0	0	0.0045
	15-29	1.72	0		1.85	0	
	30-59	36.21	10.53		35.19	0	
	≥60	62.07	89.47		62.96	94.44	
12	<15	3.64	0	0.1213	3.92	0	0.1015
	15-29	3.64	0		3.92	0	
	30-59	36.36	11.11		33.33	6.25	
	≥60	56.36	88.89		58.82	93.75	

**Figure 1:**
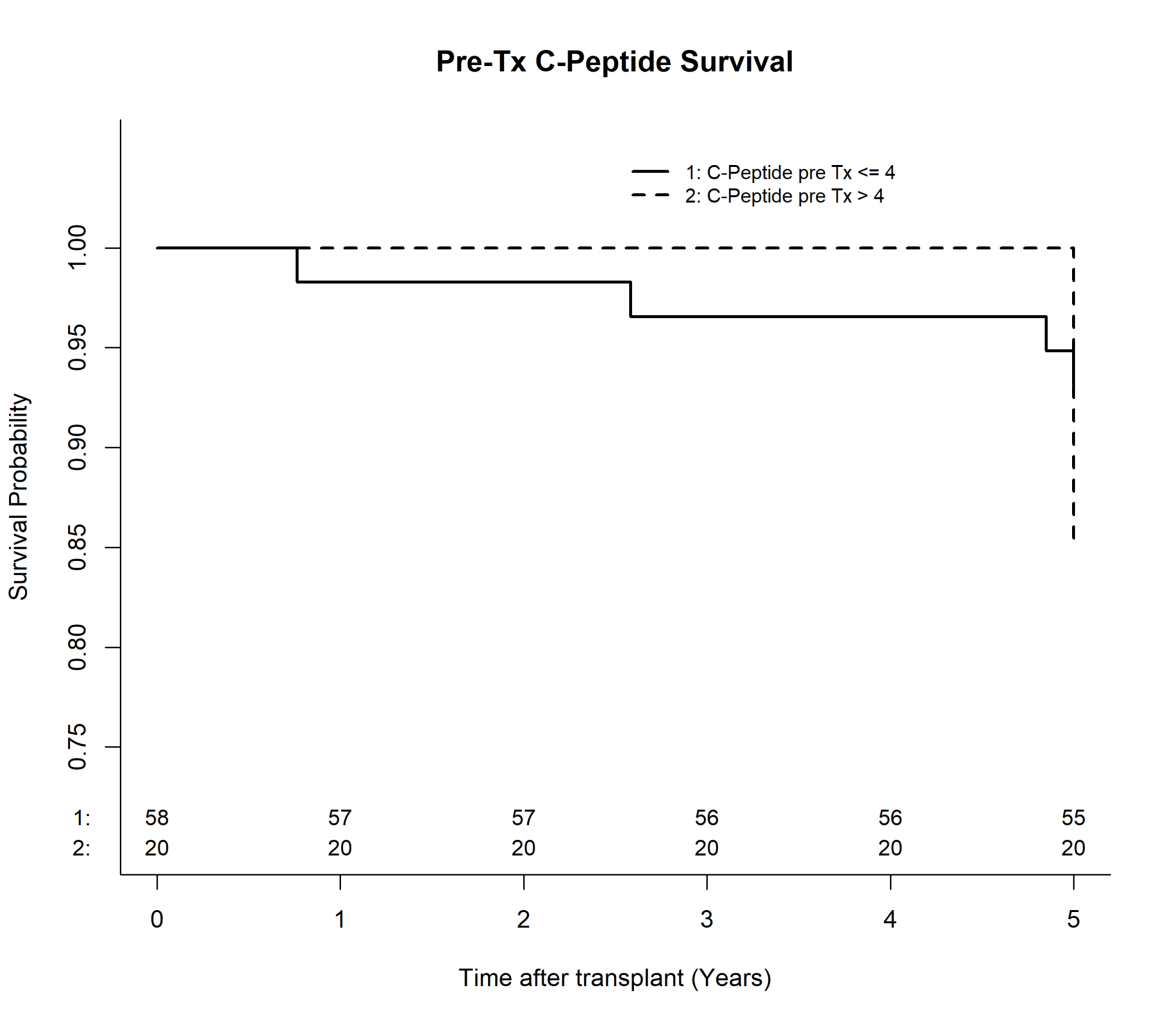
Kaplan-Meier curve comparing baseline C-peptide levels and pancreas graft survival up to five years post-transplant

Complications between the two cohorts were also collected; these were similar between the two cohorts up to 12 months post-transplant; no complications were due to technical graft loss or thrombosis (Table 4). Within the 10-year review period, the low/normal cohort versus elevated cohort had seven versus three patients with antibody mediated rejection (AMR), eight versus one with acute cellular rejection (ACR), respectively; however, only three patients in total experienced pancreas graft failure - all from the low/normal cohort. Despite the low/normal cohort experiencing more long-term complications, the difference between the two was not statistically significant. Overall, five-year patient and graft survival rates were >80%, with no statistically significant difference between the two cohorts (Figure 1). Donor characteristics were similar between the cohorts with comparable cause of death, age, sex, ethnicity/race, HbA1c, BMI, and cold ischemic time.

**Table 4:** Summary of post-transplant complications in both C-peptide cohorts up to 12 months

Complications	C-peptide pre-transplant ≤ 4 ng/mL (N=58) (number of patients)	C-peptide pre-transplant > 4 ng/mL (N=20) (number of patients)	P-value (<0.05)
Graft thrombosis	0	0	1
Readmission within three months post-transplant	44	15	1
Returned to the operating room post-transplant	1	0	1
Patients on post-tansplant insulin	0	0	1
Patients that required oral hypoglycemic agents	0	0	1
Pancreas graft losses	0	0	1
Recipient death	0	0	1

## Discussion

Normal insulin production is driven by the presence of glucose in the bloodstream. When blood glucose levels rise, β-cells in the pancreas respond by releasing both insulin and C-peptide in equimolar concentrations. This process is part of a feedback loop: increased glucose triggers insulin production, helping to maintain blood glucose levels within a healthy range. Although not directly involved in glucose regulation, C-peptide plays a crucial role in insulin’s proper folding and functionality. In individuals with pancreatic insufficiency from type 1 diabetes, there is a lack of insulin production due to the autoimmune destruction of β-cells, necessitating lifelong exogenous insulin therapy. Serum levels of C-peptide are thus expected to drop below normal levels. In individuals with acquired insulin resistance, in early disease, insulin production is increased in an attempt to compensate, eventually leading to β-cell exhaustion and dysfunction [13,14]. In both situations, individuals may meet the criteria for a pancreas transplant to achieve normoglycemic control.

After a successful pancreas transplant, insulin production is expected to be restored to near-normal levels. Given the relationship between C-peptide and endogenous insulin production, C-peptide is expected to increase significantly, indicating restoration of functionality. As such, we expect to see a reduction in HbA1c levels in these patients, indicating long-term glycemic management [13,14].

Pre-transplant patients fell within two groups: the low/normal cohort and the elevated cohort. Post-transplant, however, the elevated cohort experienced a decline in C-peptide levels, as opposed to the low/normal cohort, which experienced an increase. These data did not support our hypothesis that both groups would experience an increase in C-peptide post-transplant. This phenomenon could be attributed to factors such as altered kinetics in insulin and C-peptide processing or clearance mechanisms. For example, C-peptide may be preferentially released into the bloodstream while insulin remains intracellular within the pancreas in certain types of diabetes. Or, the body may be trying to attain homeostasis with more rapid elimination of C-peptide post-pancreas transplant in those with elevated levels. Previous studies have discussed challenges with clinical utility of C-peptide in determining the classification of diabetes [5,15]. Further studies are needed to understand the metabolism of C-peptide, especially in diabetics with type 1 diabetic morphology presumed to be insulin resistant because of their elevated C-peptide levels, and how they handle C-peptide post-transplant.

The impact of clearance was also taken into account. Primarily, C-peptide is cleared from the bloodstream through the kidneys, and about 5-10% of it is excreted unchanged in the urine [16]. In individuals in need of a pancreas transplant, impaired kidney function results in decreased clearance, contributing to elevated serum C-peptide levels despite lack of production from pancreatic failure. In contrast, an increase in kidney function post-transplant could decrease C-peptide levels despite nascent production from the newly transplanted pancreas. In our study, both cohorts saw an immediate return in kidney function at all time points post-transplant (Table 3). In the low/normal cohort, the increase in C-peptide levels can be explained by the newly restored pancreatic function, despite their improved eGFR. On the other hand, the elevated cohort also experienced a similar increase in eGFR, which should theoretically lead to increased C-peptide excretion. However, their C-peptide levels declined post-transplant, suggesting that other factors may have played a more dominant role in influencing C-peptide levels. Overall, from our analysis (Tables 2-3), nascent filtration production resulting from the newly transplanted kidney did not clearly explain the patterns observed in both cohorts. Further research is needed to explore factors beyond kidney function that might play a role in C-peptide clearance.

In addition, the differences in the number of patients on post-transplant insulin, hospital readmission rates, complication rates (both AMR and ACR), the number of patients who returned to the operating room (OR), and graft survival rates between the two cohorts were not statistically significant (Table 4). Hispanics, however, were found to be more likely to have C-peptide >4 ng/mL pre-transplant (p=0.0095) compared to their White counterparts; therefore, they could have been unnecessarily excluded from transplantation when they could have benefited (Table 1). Gender, weight, and duration of diabetes at the time of transplant were accounted for but were not significant (Table 1). Patient and graft survival at 10 years post-transplant between the cohorts were similar (both >80%; p = 0.2990), which differs from previous conversations regarding the use of pre-transplant C-peptide as a marker for transplant success [17,18].

At the 10-year follow-up, the low/normal cohort experienced three graft losses while the elevated cohort experienced no graft loss, yet this difference was not statistically significant. The grafts were considered to have failed if total insulin use was ≥ 0.5 units/kg/day for 90 consecutive days, per the current Organ Procurement and Transplantation Network (OPTN) guideline. There was no incidence of thrombosis between the two groups. Of the three graft failures, one was due to rejection secondary to non-compliance, and the other two were from the development of type 2 diabetes mellitus (T2DM). The graft failure outcomes of these two patients conflict with the 2021 Igls criteria that required HbA1C, post-transplant insulin use, C-peptide levels, and the number of severe hypoglycemic events (SHEs) return to baseline or lower before transplantation to qualify as graft failure [16]. Despite the other parameters returning or improving from pre-transplant baseline levels, C-peptide was elevated from baseline when failure was diagnosed for these two patients - from 1.49 ng/mL to 3.64 ng/mL and 1.76 ng/mL to 2.57 ng/mL. The current Igls criteria do not consider low/normal pre-transplant C-peptide patient populations who develop T2DM post-transplant and subsequently experienced graft failure. Furthermore, the proposed Igls 2.0 removes the need for comparing pre- and post-transplant C-peptide as a measure of graft failure altogether [19]. This proposed change aligns with our study's findings about the limitations of C-peptide within the graft failure definition.

This study is limited by its small sample size and single-center design, which may reduce its generalizability. Additionally, variability in C-peptide testing methods over time could affect data consistency. A follow-up period greater than 10 years could have also aided in our understanding of C-peptide trends over time. Further research is needed to fully understand the dynamics of C-peptide and insulin metabolism in patients with elevated C-peptide levels. Of particular interest is how patients with elevated pre-transplant C-peptide metabolize C-peptide in the post-transplant period and how this influences graft outcomes.

## Conclusions

This is the first study to examine the trajectory of C-peptide after pancreas transplantation. It shows a decline of C-peptide in patients with elevated levels pre-transplant, while those with low/normal levels had an increase; short and long-term patient and graft survival were similar between the two groups. The data suggest that elevated pre-transplant C-peptide levels may not be a predictor of post-transplant outcomes, as graft survival in the elevated cohort was comparable to the low/normal cohort. Our study supports placing less emphasis on C-peptide as a selection criterion for pancreas transplant candidacy. Moreover, its use in this context may lead to inequities in access, with Hispanic/Latino minorities being at a disadvantage. In individuals with low/normal pre-transplant levels that develop T2DM after a pancreas transplant, C-peptide may not offer clinical utility in the definition of graft failure in this group.
